# Modeling synthetic lethality

**DOI:** 10.1186/gb-2008-9-9-r135

**Published:** 2008-09-12

**Authors:** Nolwenn Le Meur, Robert Gentleman

**Affiliations:** 1 Fred Hutchinson Cancer Center Research, Program in Computational Biology, Division of Public Health Sciences, Fairview Avenue North, Seattle, WA 98109, USA; 2 INSERM, IRISA Symbiose, Campus de Beaulieu, 35042 RENNES Cedex, France

## Abstract

**Background:**

Synthetic lethality defines a genetic interaction where the combination of mutations in two or more genes leads to cell death. The implications of synthetic lethal screens have been discussed in the context of drug development as synthetic lethal pairs could be used to selectively kill cancer cells, but leave normal cells relatively unharmed. A challenge is to assess genome-wide experimental data and integrate the results to better understand the underlying biological processes. We propose statistical and computational tools that can be used to find relationships between synthetic lethality and cellular organizational units.

**Results:**

In *Saccharomyces cerevisiae*, we identified multi-protein complexes and pairs of multi-protein complexes that share an unusually high number of synthetic genetic interactions. As previously predicted, we found that synthetic lethality can arise from subunits of an essential multi-protein complex or between pairs of multi-protein complexes. Finally, using multi-protein complexes allowed us to take into account the pleiotropic nature of the gene products.

**Conclusions:**

Modeling synthetic lethality using current estimates of the yeast interactome is an efficient approach to disentangle some of the complex molecular interactions that drive a cell. Our model in conjunction with applied statistical methods and computational methods provides new tools to better characterize synthetic genetic interactions.

## Abstract 2

Using new computational tools in yeast, multi-protein complexes were identified that share an unusually high number of synthetic genetic interactions.

## Background

**Figure 1:**
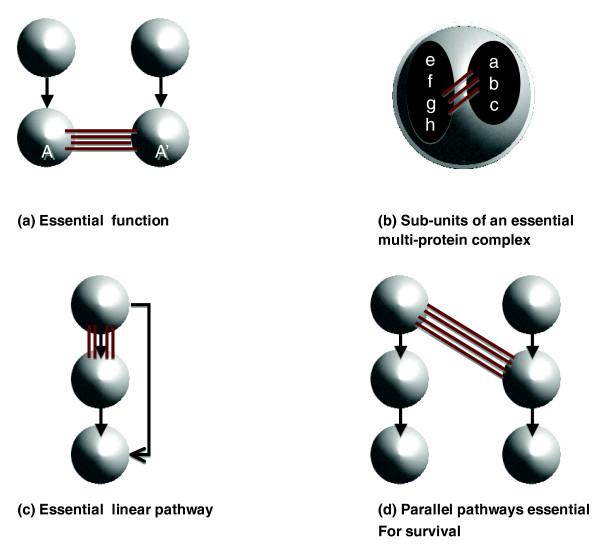
Mechanisms for synthetic genetic interactions proposed by Kaelin [1]. Each node or circle is a multi-protein complex. Synthetic genetic interactions between members of the multi-protein complexes are presented by red lines. Synthetic lethality in loss-of-function alleles can arise from at least four different mechanisms. **(a) **The cellular organizational units might be uniquely redundant with respect to an essential function; multi-protein complexes A and A' might share paralogues. **(b) **They might be two sub-units of an essential multi-protein complex: a-c form a sub-complex and synthetically interact with e-h. **(c) **They might be two interconnected components in an essential linear pathway: each mutation decreases the flow through the pathway. **(d) **They might participate in parallel pathways that are together essential: one pathway might be needed to compensate for the damage caused by mutations in the other pathway.

Two genes are said to be synthetic lethal if mutation of either alone leaves the cell viable, while simultaneous mutation leads to death. In this case, we say that one gene buffers the effect of changes in the other, that is, compensates for the effect of its deletion. The implications of synthetic lethal screening have already been discussed in the context of anticancer therapies and drug development in general [1,2]. Indeed, synthetic lethal pairs could be used to selectively kill cancer cells, but leave normal cells relatively unharmed. Although several cellular processes might give rise to synthetic lethality, none are yet well understood. Kaelin [1] proposed that synthetic lethality in loss-of-function alleles can arise from at least four different mechanisms (Figure 1). The cellular organizational units might be uniquely redundant and their roles are essential (type A; direct surrogacy), subunits of an essential multi-protein complex (type B), interconnected components in an essential linear pathway (type C), or they might participate in parallel pathways that are together essential (type D).

In *Saccharomyces cerevisiae*, previous analyses of synthetic genetic datasets have mainly relied on modeling the pairwise interactions between synthetic lethal genes. Kelley and Ideker [3] and Ulitsky and Shamir [4] concentrated on finding high density regions in graphs defined with genes as nodes and edges determined by synthetic lethal interactions, while Ye *et al. *[5] and Collins *et al. *[6] focused on identifying genes that share synthetic lethal partners. Their results are intriguing and most indicate a relationship between synthetic lethality and cellular organizational units such as multi-protein complexes or pathways. This begs the question of whether explicit modeling based on known and well established, although potentially incomplete, organizational units would be of benefit. Protein networks, based solely on pairwise interactions, cannot directly address the issues of interconnected components such as multi-protein complexes and are also unable to directly address the pleiotropic nature of many proteins.

For these reasons, we propose using an estimate of the *S. cerevisiae *interactome that uses the well established, or high confidence, protein complex predictions from Gene Ontology, the Munich Information Center for Protein Sequences (MIPs), and IntAct [7-9]. We propose a paradigm that explicitly models synthetic lethal interactions within and between cellular organizational units as suggested by Kaelin [1], with the exception of type A interactions, which are likely to rely more on sequence analysis. In this paper, we demonstrate a computational framework for identifying which and how multi-protein complexes synthetically interact, focusing on the three mechanisms (types B, C and D) suggested by Kaelin [1]. We note that there is insufficient data available to distinguish C-type interactions from D-type and we focus on assessing between and within protein complex interactions and do not try to categorize the between interactions more. We use a graph theoretic approach, although the graphs have multi-protein complexes as nodes. To illustrate our approach, we analyze the synthetic genetic array (SGA) screens reported by Tong *et al. *[10] and report in Additional data file 1 analysis of the datasets published by Pan *et al. *[11] and Collins *et al. *[6]. Finally, we compare our method with those proposed by Kelley and Ideker [3], and Ulitsky and Shamir [4] to describe the advantages and limits of our approach.

## Results and discussion

### Data quality assessment

In the absence of experimental error, most interactions between two genes (or proteins) should be symmetric, that is, gene A as a query gene interacts with the array gene B, and gene B as a query gene interacts with the array gene A. However, as shown by Chiang *et al. *[12] in yeast two-hybrid data and with affinity purification mass spectrometry experiments, the number of unreciprocated edges can be large. Chiang *et al. *[12] proposed to model the discrepancies using a binomial distribution to test the null hypothesis that, under the assumption of randomness, the number of unreciprocated in-edges and out-edges is expected to be similar for each protein. Using the same hypothesis, we assessed the symmetry in the 132 SGA data by Tong *et al. *[10] (see Materials and methods). We rejected the null hypothesis for two genes (YML032C and YCR009C) as their observed errors are probably not random (Figure S1 and Table S1 in Additional data file 1). We then removed them from further analysis.

### Synthetic genetic interactions and multi-protein complexes

In order to identify the synthetic lethal mechanisms proposed by Kaelin [1], an interactome *I *that maps genes to multi-protein complexes is required. We based our approach on curated estimates of multi-protein complexes from GO, MIPS and IntAct [7-9]. For each gene in a synthetic lethal pair, we identified all multi-protein complexes that this gene is known to be a member of. We thus reduced our interactome to the multi-protein complexes involving at least one gene of a synthetic lethal pair. In parallel, for each mutated pair, we deleted the pair if either, or both, genes were not in the interactome. Hence, only pairs where both members were also in the interactome remained. These constituted the tested edges. We then computed for any complex or pair of complexes in *I *the number of interactions tested within and between the complexes as well as the number of those interactions that were synthetic lethal. We found a maximum of 18 synthetic lethal interactions within a complex and a maximum of 49 between two multi-protein complexes, respectively.

**Figure 2:**
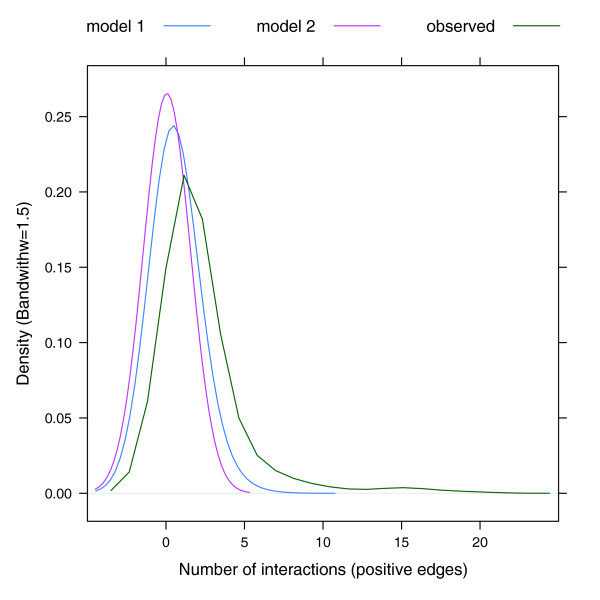
Synthetic genetic interactions are not randomly distributed in the interactome. The figure represents the distribution of the interactions (positive edges) observed within multi-protein complexes or between pairs of multi-protein complexes in Tong *et al. *[10] and in the two different permutation models. The observed data are represented by the green curve and the data derived from the permutation models are shown by the blue and pink curves. The center of the distribution for the observed data has a greater density value than those for the two simulations, meaning that synthetic genetic interactions are not randomly distributed in the interactome but rather cluster within or between pairs of multi-protein complexes.

We first tested whether or not there was a strong relationship between synthetic genetic interactions and multi-protein complex co-membership using the approach described next. We constructed a graph, *G*, whose nodes are multi-protein complexes and where edges are determined by synthetic lethal pairs as described next. For each synthetic lethal pair (*g*_1_, *g*_2_) we determine the set, *S*_1_, of multi-protein complexes that *g*_1 _is in, and the set, *S*_2_, that *g*_2 _is a member of. For any multi-protein complex *C*_i _∈ *S*_1 _and *C*_j _∈ *S*_2 _we have an edge between *C*_i _and *C*_j _in *G*. We note that if *C*_i _= *C*_j_, we have a within multi-protein complex interaction (see Materials and methods for details) and otherwise we have a between multi-protein complex interaction. Thus, one synthetic lethal pair can induce a number of interactions within and between multi-protein complexes. We corrected for the fact that many query genes were also array genes and ensured that reciprocal interactions were not counted twice. We then applied two different, but related, permutation methods to compare the observed data to randomly generated datasets (see Materials and methods). Figure 2 shows the distribution of positive edges observed within multi-protein complexes or between multi-protein complexes (green curve) compared to the distribution of the expected number of positive edges in each of the permutation models. The number of observed positive edges is globally higher than that observed for any of the simulations as the whole distribution is shifted toward higher numbers of interactions (to the right). Similar results were obtained using the genetic interaction data reported by both Pan *et al. *[11] and Collins *et al. *[6] (Figure S2 in Additional data file 1).

This reveals a strong association between multi-protein complexes and synthetic genetic interactions. In other words, this indicates that synthetic genetic interactions are associated with multi-protein complexes and are not randomly distributed in the *S. cerevisiae *interactome. It is then important to identify all multi-protein complexes, or pairs of multi-protein complexes, that possess more synthetic lethal interactions than expected by chance. For example, are the nine synthetic genetic interactions observed between the prefoldin complex [GO:0016272] and the Sec62/Sec63 complex [GO:0031207] unusual? To answer this question it is important to carefully distinguish between edges that were tested and those that were not. For instance, a maximum of 24 edges can be tested between the COMA complex [GO:0000817], composed of 6 proteins, and the prefoldin complex [GO:0016272], composed of 4 proteins. However, only 10 pairs were tested by Tong *et al. *[10] and out of those all 10 were found to synthetically interact. This is of some importance as a naive analysis would report an interaction rate of 0.41, while a more appropriate estimate of the rate is 1. We used hypergeometric distribution to test whether the number of interactions observed within and between multi-protein complexes was unusually large compared to the number of tested interactions. More precisely, for each multi-protein complex or pair of multi-protein complexes, where we had at least one positive edge, we tested whether the number of positive edges was unusually large given the hypergeometric model.

### Within versus between interactions

We observed only a small number of within multi-protein complex synthetic genetic interactions compared to between multi-protein complex interactions. However, standardized to the number of possible interactions, the number of multi-protein complexes that have within synthetic genetic interactions is large. Indeed, among the 398 multi-protein complexes of our interactome, we identified 11 multi-protein complexes that present within synthetic genetic interactions whereas 683 pairs of multi-protein complexes are linked by at least one synthetic genetic interaction. Table 1 shows that 2 of the 11 multi-protein complexes present more within synthetic genetic interactions than expected by chance (Bonferroni adjusted *P*-value ≤ 0.001). As an example, out of the 33 pairs of genes tested for interactions within the condensed nuclear chromosome kinetochore complex [GO:0000778], 19 are synthetic lethal. Similar results were observed using Pan *et al. *[11] and Collins *et al. *[6] datasets (Tables S4 and S5 in Additional data file 1). Our findings refine to the multi-protein complex level the results obtained by Tong *et al. *[10], Kelley and Ideker [3], and Ulitsky and Shamir [4]. Indeed, those authors most often identified global molecular functions (GO categories of 'molecular function' and 'biological process') for which genetic interactions between genes were surprisingly abundant. We, on the other hand, identified single multi-protein complexes with an over-abundance of synthetic lethal pairs. For example, Tong *et al. *[10] found a significant number of genetic interactions among the genes that participate in the microtubule and spindle orientation pathway, while we identify the kinetochore complex [GO:0000776] and its condensed form [GO:0000778]. In addition, we identify elements of the transcription machinery, that is, the transcription elongation factor [GO:0008023] and the Cdc73/Paf1 multi-protein complex [GO:0016593], a multi-protein complex that associates with RNA polymerase II transcription factor complexes. Kelley and Ideker [3], as well as Ulitsky and Shamir [4], searched for over-abundance of genetic interactions among pathways, which they defined as a connected set of proteins in the protein interaction network. Their pathways could be well characterized multi-protein complexes but they could also be groups of proteins from different interacting complexes (see Additional data file 1 for more details), or simply groups of proteins that interact.

**Table 1:** Multi-protein complexes with a significant number of 'within' synthetic genetic interactions (Bonferroni adjusted *P*-value < 0.001)

Complex	Odds	Expected	Size	Interact	Tested	Essential	Name
GO:0000778	161.91	0.24	33	18	33	19	Condensed nuclear chromosome k
GO:0000776	73.38	0.13	20	6	17	7	Kinetochore

Multi-protein complexes with a significant number of 'within' synthetic genetic interactions (adjusted *P*-value < 0.001). Odds, odds ratios; Expected, expected number of synthetic genetic interactions per multi-protein complex; Size, number of genes in the multi-protein complex; Interact, number of observed interactions (positive edges); Tested, number of interactions (edges) tested; Essential, total number of essential genes in the multi-protein complex; Name, full name.

Out of the 683 pairs of multi-protein complexes that share at least one synthetic genetic interaction, 86 pairs share an usually high number of synthetic genetic interactions (Bonferroni adjusted *P*-value ≤ 0.001). Table 2 presents the 10 pairs of multi-protein complexes that have the lowest adjusted *P*-values (see Table S5 in Additional data file 1 and Table S7 in Additional data file 2 for a complete listing). These results also show that not only do many pairs of multi-protein complexes present an unusual number of synthetic lethal interactions, but also that the adjusted *P*-values are extremely small. As an example, the lowest adjusted *P*-value is 5.54*e*-86 between the condensed nuclear chromosome kinetochore complex [GO:0000778] and the prefoldin complex [GO:0016272], which share 49 synthetic lethal interactions out of the 68 interactions tested. In addition, the 86 pairs of multi-protein complexes that share an usually high number of synthetic genetic interactions correspond to interactions between 53 unique multi-protein complexes. This demonstrates that some multi-protein complexes interact with more than one multi-protein complex. For instance, the SWR1 complex [GO:0000812] presents a significant number of interactions with nine other multi-protein complexes. Hence, some multi-protein complexes may be involved in more than one function, or buffering mechanism.

**Table 2:** Top 10 pairs of multi-protein complexes that present 'between' synthetic genetic interactions

						Essential genes	
						
Complex 1/complex 2	*P*-values (adjusted)	Odds	Expected	Interact	Tested	Complex 1	Complex 2
Condensed kinetochore [GO:0000778]/prefoldin [GO:0016272]	5.54e-86	350.66	0.50	49	68	19	0
Outer kinetochore [MIPS-270.20]/prefoldin [GO:0016272]	1.51e-72	Inf	0.26	35	35	12	0
Prefoldin [GO:0016272]/SWR1 [GO:0000812]	7.05e-50	326.96	0.30	29	41	0	5
Ctf3 [GO:0016272]/prefoldin [GO:0005868]	1.61e-36	674.97	0.18	20	24	0	0
Prefoldin [MIPS-270.20.20]/cytoplasmic dynein [GO:0016272]	7.23e-30	Inf	0.11	15	15	0	0
Ctf18 RFC-like [GO:0016272]/prefoldin [GO:0005869]	5.78e-27	674. 13	0.13	15	18	0	0
Prefoldin [GO:0031390]/Dynactin [GO:0016272]	5.78e-27	674. 13	0.13	15	18	4	0
Dynein motorproteins [MIPS-140.30.30.20]/prefoldin [GO:0016272]	5.78e-27	674. 13	0.13	15	18	0	0
Dynactin [MIPS-140.30.30.30]/prefoldin [GO:0016272]	5.78e-27	674. 13	0.13	15	18	0	0
Kinetochore [GO:0000776]/condensed kinetochore [GO:0000778]	3.24e-25	85.47	0.36	19	49	7	19

Top 10 pairs of multi-protein complexes that present between synthetic genetic interactions in Tong *et al*. [10]. *P*-values (adjusted), Bonferroni adjusted *P*-value of the hypergeometric test; Odds, odds ratios; Expected, expected number of synthetic genetic interactions between multi-protein complexes; Interact, number of synthetic genetic interactions observed (positive edges); Tested, number of interactions (edges) tested; Essential genes, number of essential genes in multi-protein complexes 1 and 2, respectively. Note that when all the tested interactions are synthetic genetic interactions (tested = interact) the odds ratios are infinite (Inf).

Making functional inference and explaining the mechanism involved in synthetic lethality directly from the list of synthetic lethal pairs is difficult, in part due to the pleiotropic nature of many proteins. Indeed, some proteins play a variety of functional roles in a cell and can belong to more than one multi-protein complex. Out of the 1,629 proteins that compose our interactome, 607 (37%) are in at least 2 complexes. It is then not always clear which of those functional roles and mechanisms are implicated in the effect on phenotype. Using the methods proposed here, we can address this issue to some extent. For instance, each protein of the synthetic lethal pair YDR488C-YEL061C (PAC11, CIN8, respectively) is found in two complexes. YDR488C is in the cytoplasmic dynein complex [GO:0005868] and the dynein-complex motorproteins [MIPS-140.30.30.20], while YEL061C is in the kinesin complex [GO:0005871] and the condensed nuclear chromosome kinetochore [GO:0000778]. However, our results show that only the cytoplasmic dynein complex and the kinesin complex interact more than expected by chance.

**Figure 3:**
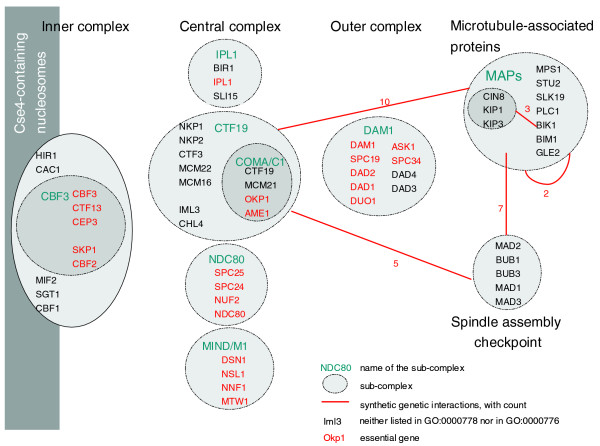
Within multi-protein complex versus between multi-protein complex synthetic lethal interactions. The kinetochore complexes are composed of sub-complexes that present many synthetic lethal interactions, especially between them. In bold are proteins that are part of either the kinetochore complex [GO:0000776] or the condensed nuclear chromosome kinetochore [GO:0000778]. In italics are the proteins that are not currently listed as part of those kinetochore complexes. The essential genes are labeled in red. The observed synthetic genetic interactions are indicated by a red line. The number associated with a red line indicates the number of synthetic genetic interactions within or between the sub-complexes. Most of the synthetic genetic interactions are between sub-complexes that contain few or no essential genes. Systematic names for these genes are available in Additional data file 1.

We also note that most of the multi-protein complexes presented in Tables 1 and 2 have a small number of essential genes. Using an approach described in our previous work [13], we found that multi-protein complexes associated with the synthetic lethal phenotype present a deficit in essential genes. However, the kinetochore complexes [GO:0000776] and [GO:0000778] seem peculiar, but interesting, as they not only have an unusual number of within and between multi-protein complex interactions (Table S3 in Additional data file 1 and Table S6 in Additional data files 1 and 2) but also contain a significant number of essential genes [13]. One explanation for these results is the intricate structure of the kinetochore complexes [14]. Indeed, the kinetochore can be divided into three sub-complexes - the inner, central, and outer complexes - some of which can be subdivided further (Figure 3). Some caution in interpretation is needed as some multi-protein complexes overlap substantially and others are not well partitioned. In the case of the kinetochore complexes, most of the sub-complexes are not described in our interactome but rather are presented as one organizational unit, the kinetochore. While some of those sub-complexes share a number of between interactions and contain few essential genes (for example, sub-unit CTF19 and the MAPs complex), others are entirely composed of essential genes and are, therefore, essential for cell viability and hence not prone to synthetic lethality (for example, NDC80 and MIND sub-complexes). In this case, the synthetic genetic interactions observed within the kinetochore complexes are likely to be between sub-unit interactions. In other words, when within multi-protein complex genetic interactions tend to cluster, it is a likely indication that there are important sub-units. We thus demonstrate that our method can help to identify interacting sub-units of an essential multi-protein complex, that is, the type B interactions proposed by Kaelin [1] (Figure 1b).

### The key role of prefoldin

Perhaps the most intriguing result is the high number of synthetic lethal interactions observed between the prefoldin complex [GO:0016272] and other multi-protein complexes; in Table 2, out of the 10 pairs of interacting multi-protein complexes, prefoldin is listed as involved in 9. Prefoldin is a heterohexameric chaperone protein believed to bind to nascent polypeptides during translation and, when synthesis is complete, deliver those unfolded polypeptides to the chaperonin TriC complex (chaperonin-containing T-complex). In *S. cerevisiae*, this multi-protein complex was shown to be especially involved in the actin and tubulin-based cytoskeleton. For instance, Lacefield and coworkers [15] reported that mutations in genes encoding components of the prefoldin complex resulted in a modest excess of beta-tubulin, and that in their *pac10*Δ mutants only 35% of tubulin was in the heterodimer form (93% in wild type). This suggests that cellular processes that require relatively rapid construction of microtubules, such as mitotic spindles biogenesis, could be substantially compromised in such a background. Furthermore, in their review, Tan *et al. *[14] clearly showed the complex interactions between the kinetochore complexes and the mitotic spindle checkpoints, which ensure normal chromosome segregation. Similarly, we show that those multi-protein complexes not only interact with prefoldin but also are tightly interconnected (Table S8 in Additional data files 1 and 2). This explains most of the observed synthetic lethal interaction between prefoldin, the kinetochore complexes, and the spindle checkpoints. Overall, this demonstrates the important role of prefoldin in mitosis.

However, all the synthetic genetic interactions observed with prefoldin cannot be explained by its ability to help in the maturation of actin and tubulin molecules. Moreover, sequence comparison of prefoldin subunits has shown that prefoldin homologs exist throughout eukarya and in the archaea [16], the later having neither actin nor tubulin. This indicates that prefoldin may also promote folding or transport of other proteins. For instance, we show that prefoldin highly interacts with the Sec62/Sec63 complex. This multi-protein complex is involved in the post-translational targeting of proteins to the endoplasmic reticulum and, to our knowledge, has not yet been described to interact with the prefoldin complex. However the Sec62/Sec63 complex is known to interact with another chaperone system, HSP70, for which the prefoldin complex can functionally substitute [17]. Hence, this example shows that our method can identify molecules that participate in parallel pathways that are together essential as proposed by Kaelin [1] (Figure 1d). Moreover, this result indicates that prefoldin can be involved in more that one cellular process, especially when cell viability is challenged.

### Complex-based approach versus pathway-based approach

A number of computational methods have been proposed to analyze genetic interactions [3-5,10,11]. Our approach is similar to the methods proposed by Kelley and Ideker [3], and Ulitsky and Shamir [4], but our methods differ in many important ways. First, we use well characterized multi-protein complexes while they propose methods to estimate pathways using protein interaction data. On one hand, using well characterized multi-protein complexes can be limiting if one is interested in gene function inference. Indeed, we are less likely to identify novel gene function as we only use genes that are already annotated to a multi-protein complex and thus to a biological process. Nevertheless, as demonstrated, using multi-protein complexes allows us to take into account the pleiotropic nature of the gene products. Moreover our approach can easily be applied to other estimates of the protein interactome. For instance, the interactome may be a mix of known and predicted multi-protein complexes, or could be the set of pathways estimated by either Kelley and Ideker [3] or Ulitsky and Shamir [4], or they could be estimated from affinity purification mass spectrometry data using methods such as those described in Scholtens *et al. *[18]. Our method could then be used for gene function prediction and identification of biological processes in which the estimated cellular organizational units are involved and interact. On the other hand, using pathway estimates as defined by Kelley and Ideker [3] or Ulitsky and Shamir [4] makes the identification of the synthetic lethal mechanisms as proposed by Kaelin [1] more difficult. Moreover, the somewhat large stochastic and systematic error rates associated with large scale protein-protein interaction data (for example, [12]) suggest that caution is needed when interpreting the results, and corroboration using well documented complexes seems prudent.

A second important difference is that we take into account the genetic interactions that were tested and the ones that were not. Kelley and Ideker [3], and Ulitsky and Sharan [4] take a different approach and estimate the prior probability of observing such interactions in the genetic interaction network (positive edges exclusively). One shortcoming of our approach is that we cannot make use of the data available in most genetic interaction databases (for example, BioGRID or MIPS) as only the observed interactions are reported and our method requires both positive and negative outputs of the genetic interaction experiments. We are thus currently limited in the number of data sources available as few high-throughput experiments have been performed and fewer have reported all genetic interactions that were tested. It is worth noting that the approach taken by the other two groups [3,4] yields biased estimates as they make the implicit assumption that all untested pairs do not interact (which is, in general, quite unlikely). In addition, taking into account all the tested interactions allows us to easily formulate a parametric statistical test such as a hypergeometric test and gain statistical power to evaluate the significance of the observed interactions.

We evaluated whether our biological findings overlap with those published by Kelley and Ideker [3], and Ulitsky and Sharan [4] (see detailed methods and results in Additional data file 1). Only Kelley and Ideker [3] investigated within-pathway relationships and we could not find any overlap with our results, indicating that the methods are complementary, at least given the current state of knowledge. This result is mainly due to the fact that we have different coverage of genetic interaction data and small overlaps between their pathways and our protein complexes (Table S11 in Additional data file 1). For the between-pathway analysis, the overlap between pairs of pathways and our pairs of multi-protein complexes is small and differs between Kelley and Ideker [3] and Ulitsky and Shamir [4] (Tables S12-S15 in Additional data file 1). This is largely due to the fact that we all use different sources of genetic interaction data. Among the pairs we have in common, we do not identify all of them as interacting more than expected by chance. This result is due to the fact that we take into account negative edges while they do not. We also found pairs of multi-protein complexes that map well to Kelley and Ideker pathways [3] that they did not detect. This is due to the lack of power of their density-based approach when the interactions are between small pathways where few interactions were tested but most of which were found to interact.

## Conclusion

In this paper, we demonstrate compared to other methods [3,4] the advantages of using current estimates of the yeast interactome, in conjunction with applied statistical methods and computational tools, to better characterize synthetic genetic interactions at the multi-protein complex level. We also show the benefit of using both positive and negative outputs of genetic interaction experiments to better estimate the role of multi-protein complexes in synthetic genetic interactions. While we applied our method to the SGA data reported by Tong and coworkers [10], our approach applies to other synthetic genetic experiments (Additional data file 1). In addition, our method can easily be applied to different estimates of organizational units within the genome, or proteome, such as groups of interacting proteins [3,4] or KEGG (Kyoto Encyclopedia of Genes and Genomes) pathways [19], and does not rely on the particular choices we have made here. In this work, we first assessed the quality of the data as it is an important aspect of the analysis of SGA datasets or any other high-throughput experiment. Typically, synthetic genetic screens are carried out by contrasting a small set of query genes with a large set of array genes, for example, 132 query genes by 4,648 array genes in Tong *et al. *[10]. One bias that must be addressed is the asymmetry of the experimental design in the sense that most often query genes are tested for interactions against most non-essential yeast genes, while for array genes only a few interactions are tested [10,20]. A second issue that should be addressed is that the query genes are typically not a simple random sample from the genome, but rather are selected from a sub-system of interest to the experimenter. Indeed, Tong *et al. *[10] were especially interested in actin based polarity, cell wall biosynthesis, microtubule based chromosome segregation, and DNA synthesis and repair. Hence, drawing inferences about all synthetic lethal interactions in yeast from these data is problematic.

We then demonstrated that the synthetic genetic interactions observed by Tong *et al. *[10] are not randomly distributed among multi-protein complexes and that some multi-protein complexes interact more than expected by chance. The same conclusions were made for the data published by Pan *et al. *[20], and Collins *et al. *[6] (Additional data file 1). We can currently identify two types of interactions: within multi-protein complex interactions and between multi-protein complex interactions. Using Tong *et al. *[10], we observed 2 multi-protein complexes with a significant number of synthetic lethal interactions and 86 pairs of multi-protein complexes (involving 1,366 interactions). However, we found that some pre-supposed within synthetic genetic interactions are actually better described as between sub-complex interactions. Indeed, Figure 3 shows that most of the synthetic lethal interactions observed within the kinetochore complexes ([GO:0000776] and [GO:0000778]) are between sub-units. We do not claim to explain all the observed interactions as, for each pair of multi-protein complexes that interact, some expertise is needed on their biological functions. Nevertheless, our methods detect two of the four synthetic lethal mechanisms proposed by Kaelin [1] (Figure 1). In particular, we found several interactions between the kinetochore sub-complexes (Figure 3) and we identified the prefoldin complex and mitosis checkpoints as parallel pathways that are together essential. Finally, our approach allows us to directly assess whether pairs of multi-protein complexes can be identified, where one multi-protein complex contains one gene of the synthetic lethal pair, while the other multi-protein complex contains the second gene of the synthetic lethal pair. We showed that this allows us to take into account the pleiotropic nature of proteins. For multi-functional genes and genes that are members of several protein complexes our approach can help to identify which of those functions is important with respect to a specific phenotype.

## Materials and methods

### Data sources

Synthetic genetic interaction data were extracted from the 132 SGA screens reported by Tong *et al. *[10] for *S. cerevisiae*. The data consist of a set of 132 query genes and 4,648 array genes, that is, 614,460 pairs of genes tested for synthetic interactions from which 4,532 were found to affect the phenotype. Tong *et al. *[10] reported synthetic lethal and synthetic sick interactions, but for simplicity we concentrate the discussion on dichotomous interpretations, that is, either some growth defect is observed or not. We also applied some filtering to reduce the dataset to pertinent genes. First, we removed the 13 essential genes [21] that were part of the SGA screens as, due to their intrinsic property of being essential, they can not explain any buffering mechanism or any of the interaction types proposed by [1]. Then, using the data quality assessment approach proposed by [12], we searched for discrepancies between the subset of the data defined by genes that were both array genes and query genes. Asymmetric results among this subset is more likely to be a false positive. We thus tested the null hypothesis that, under the assumption of randomness, the number of unreciprocated in-edges and out-edges is expected to be similar for each protein (see Additional data file 1 for more details). The alternative hypothesis was that some non-random errors exist in the data. In total, we analyzed 119 query genes and 4,648 array genes, from which 4,038 were found to affect the phenotype.

Multi-protein complex co-membership was determined from curated online databases: GO [7], MIPS [8], and IntAct [9] (see Additional data file 1 for more details). This resulted in an interactome of 398 curated multi-protein complexes and 1,629 unique genes. The full interactome is available in the R package ScISI, distributed on the Bioconductor project web site [22]. We denote the interactome *I *and the multi-protein complexes *C*_i_. The multi-protein complex names are the database ID codes with the addition of the prefix GO-, MIPS- and EBI- for the GO, MIPS, and IntAct databases, respectively.

### Computational and statistical methods

Genetic interactions can be observed within and between multi-protein complexes. If both genes (*g_1_*, *g_2_*) in the pair lie within a single multi-protein complex *C_i_*, then we use the term within multi-protein complex relationship. For every pair of multi-protein complexes, *C_i_* and *C_j_*, such that *g_1_* ∈ *C_i_* (∉ *C_j_*) and *g_2_* ∈ *C_j_* (∉ *C_i_*), we say that there is a between relationship between multi-protein complexes *C_i_* and *C_j_*. We define as tested edges within and between multi-protein complexes the pairs of genes that were tested by [10] and where both members are in the interactome *I*. For any two multi-protein complexes *C_i _*and *C_j _*in *I*, *N_ij _*denotes the number of edges tested between those two multi-protein complexes. We note as positive edges, within and between multi-protein complexes, the pairs of genes that induce a synthetic genetic interaction. Given two multi-protein complexes, *C_i _*and *C_j_*, our null hypothesis is that for any pair of genes (*g_1_*, *g_2_*), such that *g_1 _*∈ *C_i _*(∉ *C_j_*) and *g_2 _*∈ *C_j _*(∉ *C_i_*), the probability that this pair induces a synthetic genetic interaction is equal to the probability that any randomly selected pair of genes induces a synthetic genetic interaction. A similar null hypothesis was made for the case where both genes (*g_1_*, *g_2_*) are in a unique multi-protein complex.

To test this hypothesis, we used two graph theory approaches. Model 1 is synthetic genetic interaction based: we first counted the number of positive edges observed between all pairs of multi-protein complexes. We then randomly sampled the synthetic genetic pairs equal to the number reported by [10], recomputed the graph on the multi-protein complexes given these data, and finally counted the number of between edges. We did this 100 times and compared those distributions to the original distribution. Model 2 is interactome based: we applied a similar approach from the point of view of the multi-protein complexes. We first counted the number of positive edges observed between all pairs of multi-protein complexes. We then randomly sampled the gene labels of the interactome and recomputed the number of positive edges. We did this 100 times and compared those distributions to the original one. If the number of observed positive edges was greater than the simulated data we rejected the null hypothesis (see Additional data file 1 for more details).

For each multi-protein complex, or pair of multi-protein complexes, where we had at least one positive edge, we then tested whether the number of positive edges was unusually large. Then, we tested whether the multi-protein complex had more edges or whether the pairs of multi-protein complexes shared more genetic interactions than expected by chance. Towards this aim, we used a hypergeometric test (see Additional data file 1 for more details). We then adjusted the *P*-values for multiple comparisons by controlling the family-wise error rate using the Bonferroni's method. In addition, we report the raw *P*-values in Additional data file 1 as it is not clear that the false discovery rate method is the most appropriate for this analysis. Indeed, some work remains to be done to properly account for the fact that most genes are members of more than one multi-protein complex and that one multi-protein complex can have several interacting partners; hence, there is a very complex dependency between the tests.

### Availability

The data used in the statistical analysis of this paper and the algorithms developed for the proposed computational methods are all freely available as part of the SLGI and ScISI R packages, distributed on the Bioconductor project web site [22]. They are integrated into the R/Bioconductor environment for statistical computing and bioinformatics and run on multiple operating systems, including Windows, Mac OS X and Unix.

## Abbreviations

GO: Gene Ontology; MIPS: Munich Information Center for Protein Sequences; SGA: synthetic genetic array.

## Authors' contributions

NLM and RG conceived and designed the investigation. NLM performed the computational and statistical analyses. Both authors wrote the manuscript. All authors read and approved the final version of the manuscript.

## Additional data files

The following additional data are available in the online version of this paper. Additional data file 1 contains detailed information about the data sources used in the main manuscript. In the file we also present and discuss additional analysis performed in a matter of comparison with the results presented in the main paper. Additional data file 2 is an archive that contains three text-delimited files (*.csv) containing the complete results of the hypergeometric test for the between multi-protein complex analysis applied on the datasets reported by Tong *et al. *[10], Pan *et al. *[11] and Collins *et al. *[6].

## Supplementary Material

Additional data file 1 Detailed information about the data sources used in the main manuscript. We also present and discuss additional analysis performed for comparison with the results presented in the main paper. Click here for file

Additional data file 2 Each file has 13 columns: P and *P*-values (adjusted), *P*-values and adjusted *P*-values of the hypergeometric test; Odds, odds ratios; Expected,: expected number of synthetic genetic interactions between complexes; Interact (observed), number of synthetic genetic interactions observed; Tested, number of interaction tested; Essential genes, number of essential genes in complexes 1 and 2, respectively; Size, number of proteins in each complex; Names, full name of each complex. Note that when all the tested interactions are found to be synthetic genetic interactions (tested = interact), the odds ratios are infinite (Inf). Click here for file
