# Supplementary material for: Modeling synthetic lethality
Source: Genome Biol. 2008 Sep 12;9(9):R135. doi: 10.1186/gb-2008-9-9-r135 (PMC2592713; doi:10.1186/gb-2008-9-9-r135)
Supplement: Additional data file 1 — Detailed information about the data sources used in the main manuscript. We also present and discuss additional analysis performed for comparison with the results presented in the main paper. [file gb-2008-9-9-r135-S1.pdf]

Additional data file 1: Modeling synthetic lethality

Nolwenn Le Meur[1,2] *and Robert Gentleman[1]

[1]Fred Hutchinson Cancer Center Research, Program in Computational Biology,
Division of Public Health Sciences, 1100 Fairview Avenue North -
M2-B876, P.O. Box 19024, Seattle, WA 98109, USA
[2]INSERM, IRISA, Symbiose, Campus de Beaulieu, 35042 RENNES Cedex, FRANCE

This document is the supplemental file for the manuscript entitled "Modeling synthetic lethality" by LeMeur and Gentleman. This document is divided in 2 sections: data sources and results. In the first section we detail the data sources used in the main manuscript and present the data used for the additional analysis performed in a matter of comparison with the results presented in the main paper. In the results section, we present and discuss the results of our comparative analysis.

Data sources

Synthetic genetic interaction dataset

We made use of 3 different synthetic genetic interaction datasets from *Saccharomyces cerevisiae*. First, we used the 132 synthetic genetic arrays screens (SGA) reported by Tong *et al.* [10] and presented in the main manuscript. Next, we used of the data collected by Collins *et al.* [6], also using the SGA strategy, and the data issued from Pan *et al.* [11] experiment on the DNA integrity Network, using the diploid-based synthetic lethality analysis with microarray (dSLAM). The Pan *et al.* [11] dataset consists of systematic genetic interactions identified between 74 query genes and a 5641 gene-deletion set. The Collins *et al.* [6] data consists of quantitative pairwise measurements of genetic interactions between 743 genes involved in different aspects of chromosome biology. In this dataset, Collins *et al.* [6] classified as aggravating the synthetic sick or synthetic lethal interactions and alleviating the antagonist interactions. We set the aggravating interactions (Collins *et al.* S-score ≤ -5) to 1 and the other interactions (S-score ≥ -5) to 0 as we are currently only interested in synthetic lethality interactions to identify the mechanisms proposed by Kaelin [1]. For all the datasets, we removed the essential genes as published by Giaever *et al.* [21] since by definition they can not explain a buffering effect.

We performed a separate analysis for each dataset since they were obtained using different technology and were preprocessed differently.

Multi-protein complexes

Multi-protein complex co-membership was determined from curated online databases: GO [7], MIPS [8], IntAct [9]. Multi-protein complexes were extracted from GO using the Cellular Component ontology. We parsed through the Cellular Component (CC) ontology and used regular expressions to retrieve the components with the key terms 'complex', '-ase', and '-some'. The latter terms searched for multi-protein complexes such as RNA polymerase and ribosome which may not contain the term 'complex'. An analogous approach was used to parse the information from the MIPS database. The IntAct multi-protein complexes were obtained from the XML file of 'complex data' obtained from the online IntAct database [23].

The multi-protein complex co-membership data is saved as a binary matrix where rows are gene products and columns are multi-protein complexes. This matrix contains a 1 in the (i,j) position if the protein indexed

*To whom correspondence should be addressed: nlemeur@fhcrc.org

in the i-th row is a member of the protein complex of the j-th column; it contains a 0 otherwise. We note this interactome $\mathcal{I}$ and the multi-protein complexes C_i.The multi-protein complex names are the database ID codes with the addition of the prefix GO-, MIPS- and EBI- for the GO, MIPS, and IntAct databases, respectively. The interactome is currently composed of 398 curated multi-protein complexes and 1629 unique genes. It is fully available in the R package ScISI, distributed on the Bioconductor project web site [22].

R is an environment for statistical computing and bioinformatics and run on multiple operating systems including Windows, Mac OS X and Unix. To load and visualize the data in R, we first have to install the ScISI package. Next, we can load the library and the binary matrix that represents our interactome:

```
> library(ScISI)
> data(ScISIC)
> ScISIC[1:5, 1:5]

            EBI-913756 EBI-876785 EBI-852570 EBI-866976 EBI-1180400
EBI-913756           0          0          0          0           0
EBI-876785           0          0          0          0           0
EBI-852570           0          0          0          0           0
EBI-866976           0          0          0          0           0
EBI-1180400          0          0          0          0           0
```

Complex-based approach *versus* Pathway-based approach

We compared our complex-based approach with the pathway-based methods published by Kelley and Ideker [3], and Ulitsky and Shamir [4] to estimate the concordance between our approach and theirs. We begin by making a couple of general observations. First, both groups estimated pathways, using protein interaction data, and then subsequently used the pathway estimates together with (synthetic) genetic interaction data to test for enrichment of interactions (positive edges) either within (Kelley and Ideker only) or between pathways. The estimation of pathways, is in some sense a completely separate topic, and need not be mixed with the testing for enrichment of synthetic genetic interactions. We made use of established multi-protein complexes, but in fact one could take pretty much any collection of sets of genes/proteins and plug these into the inferential framework in our paper, or either of theirs.

A somewhat more important distinction between our methods is the difference on how we handle tested versus untested genetic interactions. In the notation of those papers the function $I(a, b)$ is an indicator function of whether protein $a \in V_1$ interacts with protein $b \in V_2$, where V_1 and V_2 are disjoint pathways. We all agree on which $I(a, b) = 1$, as to be observed to interact the pair must have been tested, but, we take different perspectives on assigning $I(a, b) = 0$. In our work, we require that the interaction be tested before assigning it the value zero. The approach taken by both other groups leads to bias, except in the case where all untested pairs do not interact (which is in general quite unlikely). It is important to note that this bias cannot be overcome by more data (which is what they gain, as they can use all positive reports not just those that also provide information on negative interactions), bias can only be dealt with by using appropriate models (variance is dealt with by having larger data sets).

A third distinction is on the method of inference used. Again, from our perspective the differences are not large. In the models used by Kelley and Ideker [3], and Ulitsky and Shamir [4], the parameter β is never estimated, and rather a value is arbitrarily assumed. If one considers all tested genetic interactions and then the proportion of them that were positive, this would be an estimate of the parameter $r(a, b)$ in their formulae, and a similar estimate of β can be made for the comparison of any specific set of pathways. We noted, that equipped with such an estimate one can easily formulate a Hypergeometric test or other explicit model. This formulation has many advantages such as allowing for the natural incorporation of covariates. These tests are also useful for comparison to the permutation-type models proposed by the other two groups, as large discrepancies between the two may be informative. We also proposed and used a permutation test. The main difference is that our tests reflect the difference between edges that were tested and those that were not, as discussed in the previous paragraph, and it is there that the largest differences between the

approaches are manifested.

We observe that we use a substantially different definition for protein-protein interaction than either of those sets of authors. Kelley and Ideker [3] define as pathway a densely connected set of proteins in the protein interaction network, and a 'between-pathway model' as a disjoint pair of pathways that are densely interconnected in the genetic interaction network. Ulitsky and Shamir [4] choose a broader definition for pathway and define it to be a connected sub-network in the protein interaction network. In both analysis, a pathway might then be a well characterized multi-protein complex but it could also be a group of proteins from different interacting complexes. In contrast, we use known multi-protein complexes extracted from curated online databases and investigate genetic interactions within one multi-protein complex or between a pair of multi-protein complexes.

We also remark that all authors used different sources of protein interaction network and genetic interaction data which limit the comparisons. Kelley and Ideker [3] used protein interaction data from the DIP database [24], containing 15429 protein-protein interactions, 5869 protein-DNA regulatory interactions and 6306 shared-reaction metabolic relationships. Ulitsky and Shamir [4] used protein interaction network consisting of protein-protein and protein-DNA interactions from multiple undocumented sources and contained 68172 interactions covering 6184 proteins. We made use of multi-protein complexes extracted from curated online-databases (see *Multi-protein section* for more details) For synthetic genetic interactions, Kelley and Ideker [3] used the data reported by Tong *et al.* [10] and the MIPS database [25]. Ulitsky and Shamir [4] used the BioGRID database [26]. We made used of the data reported by Tong *et al.* [10] for this comparison.

We downloaded the pathway data from the online supplementary material [3.4]. They define a pathway as a list of genes and no explicit annotation (name) is available. Kelley and Ideker [3] give an arbitrary identification number for each pathway and give a second arbitrary identification number for each between-pathway model. Ulitsky and Shamir [4] only give arbitrary identification number for each between-pathway model.

For the between models, we analyzed their data in 2 different ways: from their pair of pathways to our multi-protein complexes and from our interacting protein complexes to their pathways.

For the first approach, for each pair of pathways they found to interact, let's say (P_n,P_m), we searched if they mapped some of our interacting protein complexes, *e.g.*, (C_i, C_j). We worked on the between-pathway interactions basis and apply the same procedure for each pair of pathways. First, we verified that their genes were listed in our genetic interaction data and in our interactome. Second, we mapped both pathways of a pair to our multi-protein complexes. In other words, we search if all the genes of P_n are in C_k. We note that most often one pathway may overlap with more than one multi-protein complex as their pathway are usually smaller than our multi-protein complexes (in that case we take into account all the complexes identified). While looking at a pair of pathways, we found 3 cases: (i) no pathway matched a multi-protein complex, (ii) only one of the two pathways overlapped with a multi-protein complex (P_n overlaps with C_i but P_m do not overlap with any of our multi-protein complexes), and (iii) the two pathways mapped to at least one multi-protein complex. We thus only kept the pathway pairs from case (iii), where both pathways mapped to at least one multi-protein complex (P_n overlaps with C_i and P_m with C_j). We then determined whether the associated pairs of multi-protein complexes (C_i, C_j) shared synthetic genetic interactions, and, if they did, whether the number of synthetic genetic interactions were unusually high. We note that we applied the same procedure per pathway in the within models, investigated by Kelley and Ideker [3].

For the second approach, we searched if both members of our pairs of interacting multi-protein complexes (C_i, C_j) mapped one (or more) of their pathways, *e.g.*, P_u and P_v. Here, for one multi-protein complex, we searched if at least half of its genes map one (or more) of their pathways (as they are smaller in size). Then, we searched if the authors found those pathways interacting and investigated why they did or did not interact.

Computational and statistical paradigm

Synthetic genetic array quality assessment

As proposed by Chiang *et al.* [12], we used a Binomial distribution to model the discrepancies between the subset of the data defined by genes that are both array genes and query genes. We tested the null hypothesis that, under the assumption of randomness, the number of unreciprocated in-edges and out-edges is expected to be similar for each protein. In other words, in a graph where each node is a protein, a protein A should randomly interact with X proteins (out-edges) as many times as Y other proteins randomly interact with protein A (in-edges). The alternative hypothesis is that some non-random errors exist in the data. We adjusted the P-values for multiple testing by controlling the false discovery rate using the method described by Benjamini and Hochberg [27]. We then defined as problematic the genes with a P-value ≤ 0.01.

Permutation models

Two approaches can be used to assess the significance of the observed data using permutational (resampling) approaches. We briefly describe both, and then provide details of the simulations. The null hypothesis is that there is no association between synthetic genetic interactions and the fact that one protein is in C_i while the other is in C_j.

In the first approach we based the resampling on the synthetic genetic pairs. For the observed data, we reduced the interactome to those multi-protein complexes that contained at least one protein that was tested in the synthetic genetic screen. And we must reduce the set of synthetic genetic pairs to those where both members were in the reduced interactome, since otherwise we would not have observed an edge and the pair would have been uninformative. We denoted those pairs N_{red}. Next we considered the whole set of proteins contained in the reduced interactome. From these we selected, at random, N_{red} pairs. These induced a set of edges between all pairs of complexes in the reduced interactome. We repeated this procedure, n times, so that at the end, for every pair of complexes there were n values that corresponded to the number of synthetic genetic edges for each simulation. The observed number of edges was then compared to this distribution and significance assessed.

The second approach considered the problem from the point of view of the multi-protein complexes. Here, we kept the set of synthetic genetic pairs fixed, but permuted the labels of the genes for the protein complexes. For each permutation, we again computed the number of edges between every pair of complexes. Thus, again after n permutations we compared the observed number of synthetic genetic edges to the set of simulated values and assessed significance. This second approach is a bit unusual, as the null hypothesis more directly reflects a belief that there is some other set of multi-protein complexes of the same size and with the same sorts of interactions within and between multi-protein complexes.

Hypergeometric tests

For each multi-protein complex or pair of multi-protein complexes, where we had at least one positive edge (*i.e.*, one synthetic genetic interaction), we tested whether or not the number of positive edges was unusually large.

Under the null hypothesis there is no relationship between synthetic genetic interaction and the fact that the edge is between complexes C_i and C_j. For a single pair of complexes (C_i,C_j) the appropriate distribution is the Hypergeometric. In this case we consider an urn that contains one ball for each edge tested for synthetic lethality between complexes. These balls are either white or black, depending on whether the tested pair of proteins was in fact synthetic lethal or not. More precisely, for any pair of complexes, the four parameters of the Hypergeometric distribution are: N the total number of balls or the total number of between complex edges that were tested for synthetic lethality; m the number of black balls or the total number of edges that were synthetic lethal, l the number of balls drawn or the number of tested edges between complexes C_i and C_j; and k the number of black balls drawn or the number of synthetic lethal edges between complexes C_i and C_j. We also note that the number of synthetic genetic edges approximately follows a Hypergeometric

distribution as there is a small amount of correlation in the data. Indeed, two proteins typically generate a number of edges, since one or both are often in several multi-protein complexes.

Finally, we adjusted the for multiple comparisons by controlling the family wise error rate using the Bonferroni's method. We set a threshold at 0.001 and the multi-protein complexes or pairs of multi-protein complexes with an associated P-values ≤ 0.001 are said to share more genetic interactions than expected by chance.

Results

Data quality assessment

Figure S1 presents the results of testing the hypothesis that the distribution of the synthetic genetic interaction screens follows a Binomial distribution. Panel (A) shows the results obtained from the synthetic genetic array screens by Tong *et al.* [10], Panel (B) from the diploid-based synthetic lethality analysis with microarray by Pan [11], and Panel (C) from the synthetic genetic array screens by Collins *et al.* [6]. For panel (A) and (B), the left hand side of each density plot shows some outliers. It seems that only a couple of genes present some bias. The data in Panel (C) are peculiar as no outlier is detected and all P-values $= 1$. It demonstrates that Collins and co-workers (2007) heavily preprocessed their data before releasing them in the public domain. Table S1 and Table S2 list the number of reciprocated and unreciprocated edges for Tong's and Pan's data, showing that the majority of the genes that were both array and query genes found each other (see additional files for complete listing). We conclude in that case that both experiments, Tong *et al.* [10] and Pan *et al.* [11], are of even quality and only a few gene interactions seem questionable.

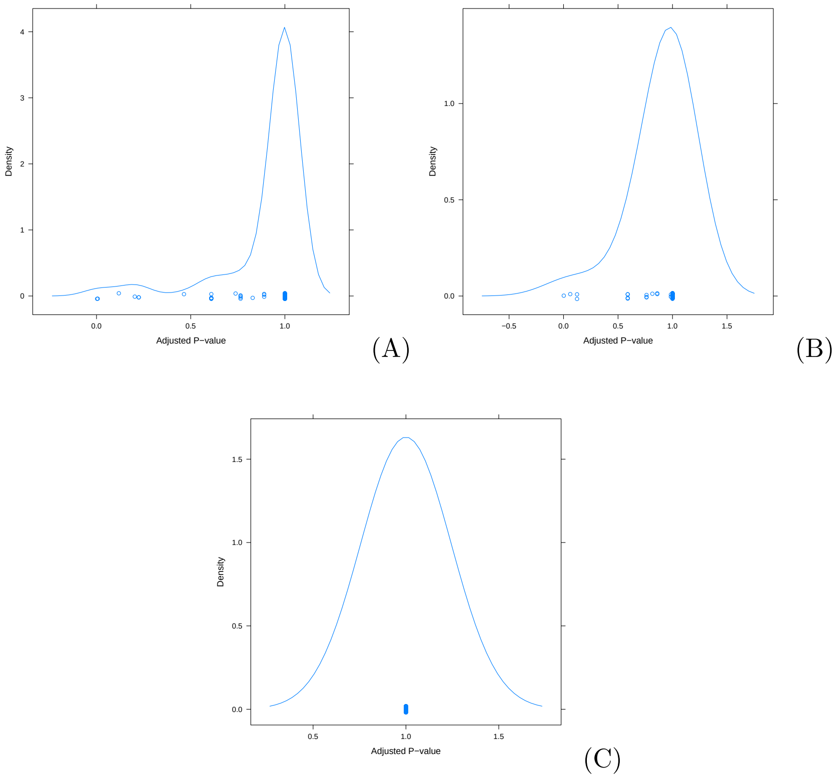

Figure S1: Assessment of the symmetry in synthetic genetic interactions datasets. Smooth density of the proportion of reciprocal interaction for the genes that were both array genes and query genes. Panel (A) SGA data from Tong *et al.* (2004); Panel (B) dSLAM data from Pan *et al.* (2006); Panel (C) SGA screens from Collins *et al.* (2007).

	RE	URO	URI	P-value	P-value (adjusted)
YCR009C	2	16	0	3.05e−05	3.17e−03
YML032C	5	0	14	1.22e−04	6.35e−03
YHR030C	12	1	12	3.42e−03	1.18e−01
YER007W	9	0	8	7.81e−03	2.03e−01
YNL298W	16	2	12	1.29e−02	2.24e−01
YDR388W	6	12	2	1.29e−02	2.24e−01
YJL092W	12	6	0	3.12e−02	4.64e−01
YLR418C	4	5	0	6.25e−02	6.09e−01
YER173W	3	0	5	6.25e−02	6.09e−01
YOR058C	10	9	2	6.54e−02	6.09e−01

Table S1: Assessing the symmetry of genetic interaction found by Tong et al. (2004), for genes that are both array genes and query genes. RE: Reciprocated edges are pair of genes that find each other; URO and URI: Unreciprocated out- and in-edges the number of unreciprocated edges found when the gene is as a query or a array genes, respectively; P-value (adjusted): Benjamini-Hochberg adjusted P-values.

	RE	URO	URI	P-value	P-value (adjusted)
YDL162C	25	16	0	3.05e−05	2.04e−03
YDR439W	23	1	13	1.83e−03	6.13e−02
YCR086W	21	2	13	7.39e−03	1.24e−01
YOR209C	21	2	13	7.39e−03	1.24e−01
YBR278W	1	0	5	6.25e−02	5.89e−01
YMR048W	31	5	0	6.25e−02	5.89e−01
YJL092W	17	14	5	6.36e−02	5.89e−01
YOR144C	26	1	7	7.03e−02	5.89e−01
YJL047C	8	8	2	1.09e−01	7.61e−01
YGL020C	30	1	6	1.25e−01	7.61e−01

Table S2: Assessing the symmetry of genetic interaction found by Pan et al. (2006), for genes that are both array genes and query genes. RE: Reciprocated edges are pair of genes that find each other; URO and URI: Unreciprocated out- and in-edges the number of unreciprocated edges found when the gene is as a query or a array genes, respectively; P-value (adjusted): Benjamini-Hochberg adjusted P-values.

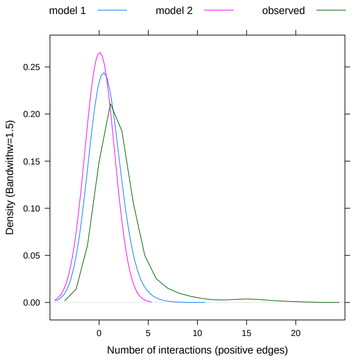

(A)

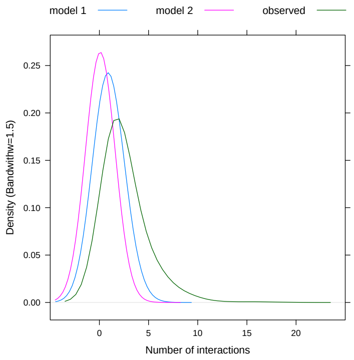

(B)

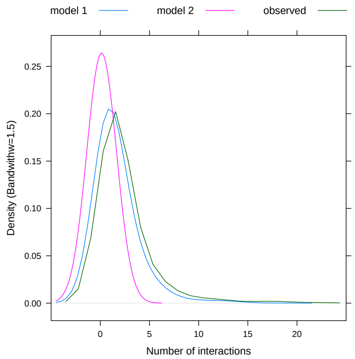

(C)

Figure S2: Synthetic genetic interactions are not randomly distributed in the interactome. Panel (A) SGA data from Tong *et al.* (2004). Panel (B) dSLAM data from Pan *et al.* (2006). Panel (C) SGA screens from Collins *et al.* (2007). In each panel is represented the distributions of the positive edges observed within multi-protein complex or between pair of complexes compare to the 2 different permutation models. In green are the observed data, in blue and pink are the data issued from the permutation models.

Synthetic genetic interactions and protein complexes

For data reported by Pan *et al.* [11], and as described for the Tong *et al.* [10] data, we found that synthetic genetic interactions within multi-protein complex or between pair of multi-protein complexes are not randomly distributed (Figure S2 Panel B). However, this is questionable for the data reported by Collins *et al.* [6] when comparing the observed interactions and the one expected the first model (Figure S2 Panel C). This result might again be explained by the fact that Collins *et al.* [6] heavily preprocessed their data.

The next question of interest was then whether those multi-protein complexes possess more synthetic lethal pairs than expected by chance. Using an Hypergeometric test, we found indeed that many multi-protein complex or pairs of multi-protein complexes have a surprisingly large number of positive edges. Table S3 to Table S5 show the multi-protein complexes that present *within* synthetic lethal interactions in Tong *et al.* [10], Pan *et al.* [11], and Collins *et al.* [6], respectively.

We found that in the data published by Pan *et al.* [11] the number of protein complex where we detected within synthetic genetic interactions is small and none of those complexes presented more interaction than expected by chance (Table S4). This result is mostly due to the small size of the query gene set (only 74 genes). The results obtained by Collins *et al.* [6] for the condensed nuclear chromosome kinetochore (GO:0000778) confirm our first results on Tong *et al.* [10] dataset. Although the result for the kinetochore (GO:0000776) is different, fewer interactions were tested.

Complex	P-value (adjusted)	Odds	Expected	Size	Interact	Tested	Essential	Name
GO:0000778	0.00e+00	161.91	0.24	33	18	33	19	condensed nuclear chromosome k...
GO:0000776	0.00e+00	73.38	0.13	20	6	17	7	kinetochore
GO:0008023	5.90e−02	44.81	0.09	19	3	12	4	transcription elongation facto...
MIPS-190.10	1.13e−01	268.79	0.02	3	2	3	0	1,3-beta-D-glucan synthase
GO:0016593	2.25e−01	134.39	0.03	7	2	4	0	Cdc73/Paf1 complex
GO:0000118	1.00e+00	13.44	0.24	18	3	33	0	histone deacetylase complex
GO:0000136	1.00e+00	33.59	0.04	6	1	5	0	alpha-1,6-mannosyltransferase ...
GO:0000148	1.00e+00	Inf	0.01	3	1	1	1	1,3-beta-glucan synthase compl...
GO:0005871	1.00e+00	26.88	0.09	6	2	12	0	kinesin complex
GO:0030008	1.00e+00	134.36	0.01	10	1	2	7	TRAPP complex
GO:0000133	1.00e+00	44.79	0.03	5	1	4	0	polarisome

Table S3: Multi-protein complexes with within synthetic genetic interactions in Tong *et al.* (2004). Adjusted P-value: Bonferroni adjusted P-value of the Hypergeometric test; Expected: expected number of synthetic genetic interactions per the multi-protein complex; Odds: odds ratios; Size: number of genes in the multi-protein complex; Essential: total number of essential genes in the multi-protein complex. Name: full name. Note that for a multi-protein complex when all the genes tested for synthetic genetic interactions present an interaction (Observed = Size) the odds ratio are infinite (Inf).

Complex	P-value (adjusted)	Odds	Expected	Size	Interact	Tested	Essential	Name
GO:0000502	1.00	12.05	0.44	11	4	15	1	proteasome complex
GO:0005678	1.00	33.12	0.06	3	1	2	0	chromatin assembly complex
GO:0000123	1.00	11.04	0.12	9	1	4	2	histone acetyltransferase comp...
GO:0035267	1.00	8.28	0.15	13	1	5	5	NuA4 histone acetyltransferase...
MIPS-360	1.00	3.31	0.32	36	1	11	28	Proteasome

Table S4: Synthetic genetic interaction within multi-protein complexes in Pan *et al.* (2006). Adjusted P-value: Bonferroni adjusted P-value Odds: odds ratios; Expected: expected number of synthetic genetic interaction within complex; Size: size of the complex; Interact (observed): number of synthetic genetic interactions observed; Tested: number of interaction tested; Essential.: number of essential genes in the complex; Name: full names of the complex.

Complex	P-value (adjusted)	Odds	Expected	Size	Interact	Tested	Essential	Name
GO:0000778	0.00	14.58	2.24	33	21	55	19	condensed nuclear chromosome k...
GO:0000118	0.04	4.29	3.71	18	14	91	0	histone deacetylase complex
MIPS-510.40	0.13	3.75	4.16	35	14	102	20	RNA polymerase II holoenzyme
MIPS-510.40.20	0.41	4.45	2.57	21	10	63	10	Kornberg's mediator (SRB) comp...
GO:0000776	0.44	11.78	0.61	20	5	15	7	kinetochore
MIPS-360	0.87	15.70	0.41	36	4	10	28	Proteasome
EBI-1252323	1.00	11.77	0.24	13	2	6	8	Anaphase-Promoting Complex var...
EBI-1252490	1.00	11.77	0.24	14	2	6	9	Anaphase-Promoting Complex var...
EBI-1252538	1.00	11.77	0.24	14	2	6	8	Anaphase-Promoting Complex var...
EBI-1252572	1.00	11.77	0.24	14	2	6	8	Anaphase-Promoting Complex var...
EBI-1251060	1.00	2.88	2.24	20	6	55	5	SAGA complex
GO:0000119	1.00	3.66	2.12	20	7	52	10	mediator complex
GO:0000502	1.00	2.83	1.14	11	3	28	1	proteasome complex
GO:0000508	1.00	0.44	2.24	12	1	55	0	Rpd3L complex
GO:0005678	1.00	15.70	0.20	3	2	5	0	chromatin assembly complex
GO:0005680	1.00	11.77	0.24	16	2	6	9	anaphase-promoting complex
GO:0005732	1.00	11.77	0.12	22	1	3	17	small nucleolar ribonucleoprot...
GO:0005871	1.00	5.89	0.41	6	2	10	0	kinesin complex
GO:0005956	1.00	11.77	0.24	4	2	6	0	protein kinase CK2 complex
GO:0008023	1.00	1.08	3.71	19	4	91	4	transcription elongation facto...
GO:0016593	1.00	2.62	0.41	7	1	10	0	Cdc73/Paf1 complex
GO:0046540	1.00	23.55	0.24	32	3	6	22	U4/U6 x U5 tri-snRNP complex
GO:0046695	1.00	2.48	0.86	16	2	21	5	SLIK (SAGA-like) complex
GO:0000788	1.00	1.39	1.47	11	2	36	2	nuclear nucleosome
MIPS-230.20.20	1.00	7.36	0.86	16	5	21	5	SAGA complex
MIPS-320	1.00	2.48	0.86	8	2	21	1	Nucleosomal protein complex
MIPS-120	1.00	5.89	0.41	8	2	10	1	Casein kinase

Table S5: Synthetic genetic interaction within multi-protein complexes in Collins *et al.* (2007). Adjusted P-value: Bonferroni adjusted P-value of the Hypergeometric test; Odds: odds ratios; Expected: expected number of synthetic genetic interaction within complex; Size: size of the complex; Interact (observed): number of synthetic genetic interactions observed; Tested: number of interaction tested; Ess.: number of essential genes in the complex; Name: full names of the complex.

The main effect is again the high number of *between* multi-protein complexes synthetic genetic interactions. Table S6 to Table S8 show the top 10 multi-protein complexes that present *between* synthetic lethal interaction in Tong *et al.* [10], Pan *et al.* [11], and Collins *et al.* [6], respectively. See additional data file 2 (*.cvs files) for complete listing.

Complex 1 - Complex 2	P-values (adjusted)	Odds	Expected	Interact	Tested	Essential Genes Complex 1	Essential Genes Complex 2
GO:0000778–GO:0016272	5.54e−86	350.66	0.50	49	68	19	0
MIPS-270.20–GO:0016272	1.51e−72	Inf	0.26	35	35	12	0
GO:0016272–GO:0000812	7.05e−50	326.96	0.30	29	41	0	5
GO:0016272–GO:0005868	1.61e−36	674.97	0.18	20	24	0	0
MIPS-270.20.20–GO:0016272	7.23e−30	Inf	0.11	15	15	0	0
GO:0016272–GO:0005869	5.78e−27	674.13	0.13	15	18	0	0
GO:0031390–GO:0016272	5.78e−27	674.13	0.13	15	18	4	0
MIPS-140.30.30.20–GO:0016272	5.78e−27	674.13	0.13	15	18	0	0
MIPS-140.30.30.30–GO:0016272	5.78e−27	674.13	0.13	15	18	0	0
GO:0000776–GO:0000778	3.24e−25	85.47	0.36	19	49	7	19

Table S6: Top 10 pairs of multi-protein complexes that share between synthetic genetic interactions in Tong *et al.* (2004). P-value (adjusted) : Bonferroni adjusted P-value of the Hypergeometric test; OddsRatio: odds ratios; expected: expected number of synthetic genetic interaction between multi-protein complexes; tested: number of interaction tested; interact (observed): number of synthetic genetic interactions observed; Essential: number of essential genes in complex 1 and 2, respectively; Note that when all the tested interactions are found synthetic genetic interactions (tested = interact) the odds ratio are infinite (Inf).

Complex 1 - Complex 2	P-values (adjusted)	Odds	Expected	Interact	Tested	Essential Genes Complex 1	Essential Genes Complex 2
GO:0031390–GO:0016272	5.61e−20	265.68	0.53	16	18	4	0
GO:0000778–GO:0031390	3.80e−16	31.56	1.14	19	39	19	4
GO:0000417–EBI-1236334	5.30e−16	Inf	0.35	12	12	0	0
GO:0043529–GO:0008023	1.56e−15	53.13	0.76	16	26	0	4
MIPS-220–GO:0043529	8.44e−15	44.28	0.82	16	28	0	0
GO:0043529–GO:0000508	1.16e−13	58.09	0.64	14	22	0	0
GO:0043529–GO:0017119	6.19e−13	Inf	0.29	10	10	0	3
GO:0033263–GO:0031390	8.35e−12	66.37	0.53	12	18	0	4
GO:0030870–EBI-1236334	2.12e−11	Inf	0.26	9	9	0	0
GO:0031390–EBI-1236334	2.12e−11	Inf	0.26	9	9	4	0

Table S7: Top 10 pairs of multi-protein complexes that share between synthetic genetic interactions in Pan *et al.* (2006). P-values (adjusted): Bonferroni adjusted P-value of the Hypergeometric test; OddsRatio: odds ratios; Expected: expected number of synthetic genetic interaction between complexes; Tested: number of interaction tested; Interact (observed): number of synthetic genetic interactions observed; Essential Genes: number of essential genes in complex 1 and 2, respectively; Note that when all the tested interactions are found synthetic genetic interactions (tested = interact) the odds ratio are infinite (Inf).

Complex 1 - Complex 2	P-values (adjusted)	Odds	Expected	Interact	Tested	Essential Genes Complex 1	Essential Genes Complex 2
GO:0000812–GO:0000508	9.23e−49	37.66	3.59	54	88	5	0
GO:0016272–GO:0000812	2.28e−43	71.03	2.28	42	56	0	5
GO:0000812–GO:0000118	1.58e−34	18.43	4.56	49	112	5	0
GO:0000778–GO:0016272	1.63e−27	21.88	3.14	37	77	19	0
MIPS-240.20–GO:0000812	2.46e−19	89.69	0.98	19	24	0	5
GO:0000776–GO:0000778	2.59e−14	14.53	2.57	24	63	7	19
GO:0046540–GO:0000812	4.71e−14	30.34	1.30	18	32	22	5
GO:0043529–GO:0008023	5.82e−11	27.22	1.14	15	28	0	4
GO:0032045–GO:0000812	5.97e−11	70.74	0.65	12	16	4	5
MIPS-510.190.150–GO:0000812	5.97e−11	70.74	0.65	12	16	0	5

Table S8: Top 10 pairs of multi-protein complexes that share between synthetic genetic interactions in Collins *et al.* (2007). P-value (adjusted): adjusted P-value of the Hypergeometric test; Odds: odds ratios; Expected: expected number of synthetic genetic interaction between complexes; Tested: number of interaction tested; Interact: number of synthetic genetic interactions observed; Ess1 and Ess2: number of essential genes in complex1 and 2, respectively; Note that when all the tested interactions are found synthetic genetic interactions (tested = interact) the odds ratio are infinite (Inf).

Table S9 lists the multi-protein complexes that significantly interact with at least one other multi-protein complex in one the datasets. In total, 95 multi-protein complexes are involved in some synthetic genetic interaction in at least one of the 3 research group. This analysis also shows that 69 were found in the 3 groups, 10 multi-protein complexes were found both in Tong *et al.* [10] and Pan *et al.* [11], 5 in both Tong *et al.* [10] and Collins *et al.* [10], and 1 in both Pan *et al.* [11] and Collins *et al.* [6]. Finally, 1 multi-protein complexes were only found when looking at Collins *et al.* [6] dataset, 5 at Pan *et al.* [11], and 4 at Tong *et al.* [10].

Table S9: Description of the multi-protein complexes that present a significant number of 'between' synthetic genetic interactions with at least one other protein complex and in at least one of the experiments by Tong et al. (2004), Pan et al. (2006) or Collins et al (2007).

Complex	Name	Experiments
GO:0000778	condensed nuclear chromosome kinetochore	T, P, C
GO:0016272	prefoldin complex	T, P, C
MIPS-270.20	Outer Kinetochor Protein Complex	T, P, C
GO:0000812	SWR1 complex	T, P, C
GO:0005868	cytoplasmic dynein complex	T, P, C
MIPS-270.20.20	Ctf3 protein complex	T, P, C
GO:0005869	dynactin complex	T, C
GO:0031390	Ctf18 RFC-like complex	T, P, C
MIPS-140.30.30.20	Dynein-complex motorproteins	T, P, C
MIPS-140.30.30.30	Dynactin complex	T, C
GO:0000776	kinetochore	T, P, C
GO:0000508	Rpd3L complex	T, P, C
GO:0008023	transcription elongation factor complex	T, P, C
GO:0005871	kinesin complex	T, P, C
GO:0000817	COMA complex	T, P, C
GO:0000942	outer kinetochore of condensed nuclear chromosome	T, P, C
MIPS-270.20.10	Ctf19 protein complex	T, P, C
GO:0031207	Sec62/Sec63 complex	T, P, C
GO:0016593	Cdc73/Paf1 complex	T, P, C
GO:0000118	histone deacetylase complex	T, P, C
MIPS-520.10	ER protein-translocation complex (Sec complex)	T, P, C
GO:0048476	Holliday junction resolvase complex	T, P, C
GO:0031422	RecQ helicase-Topo III complex	T, P, C
GO:0030904	retromer complex	T, P
MIPS-240.20	HDB complex	T, P, C
MIPS-510.40.10	RNA polymerase II	T, P, C
GO:0000136	alpha-1,6-mannosyltransferase complex	T, P, C
GO:0045298	tubulin complex	T, C
GO:0033062	Rhp55-Rhp57 complex	T, P, C
MIPS-510.40	RNA polymerase II holoenzyme	T, P, C
GO:0000123	histone acetyltransferase complex	T, P, C
MIPS-260.30.30.10	Vps35/Vps29/Vps26 complex	T
GO:0005678	chromatin assembly complex	T, P, C
GO:0030870	Mre11 complex	T, P, C
MIPS-260.20	Clathrin-associated protein (AP) complex	T, P, C
GO:0032045	guanyl-nucleotide exchange factor complex	T, P, C

Continued on next page

Complex	Name	Experiments
MIPS-360	Proteasome	T, P, C
GO:0031251	PAN complex	T, P
GO:0017119	Golgi transport complex	T, P, C
GO:0000502	proteasome complex	T, P, C
GO:0031417	NatC complex	T, P
GO:0033551	monopolin complex	T, P, C
GO:0005671	Ada2/Gcn5/Ada3 transcription activator complex	T, P, C
GO:0016602	CCAAT-binding factor complex	T
EBI-866976	DNA polymerase delta heterotrimer	T, P, C
GO:0042719	mitochondrial intermembrane space protein transporter complex	T, C
GO:0031391	Elg1 RFC-like complex	T, P, C
GO:0043529	GET complex	T, P, C
GO:0008290	F-actin capping protein complex	T
GO:0031371	ubiquitin conjugating enzyme complex	T, C
GO:0045009	chitosome	T
GO:0000509	Rpd3S complex	T, P, C
GO:0035267	NuA4 histone acetyltransferase complex	T, P, C
GO:0000417	HIR complex	T, P, C
EBI-1236334	chromatin assembly factor 1	T, P, C
MIPS-220	H+-transporting ATPase, vacuolar	T, P, C
GO:0033263	CORVET complex	T, P, C
GO:0000814	ESCRT II complex	P
GO:0032806	carboxy-terminal domain protein kinase complex	T, P
MIPS-260.80	Class C Vps protein complex	P
GO:0000221	vacuolar proton-transporting V-type ATPase, V1 domain	T, P, C
GO:0030897	HOPS complex	T, P
GO:0031415	NatA complex	T, P, C
GO:0005830		T, P, C
MIPS-370	Protein N-acetyltransferase	T, P, C
GO:0048188	COMPASS complex	T, P, C
GO:0005838	proteasome regulatory particle	T, P, C
GO:0000274	mitochondrial proton-transporting ATP synthase, stator stalk	P
GO:0030015	CCR4-NOT core complex	P
MIPS-125.10.10	Ddc1p-Mec3p complex	T, P, C
MIPS-510.190.110	CCR4 complex	T, P
MIPS-420.50	F0/F1 ATP synthase (complex V)	T, P
GO:0000815	ESCRT III complex	T, P
GO:0000938	GARP complex	T, P
GO:0005667	transcription factor complex	T, P, C
GO:0046540	U4/U6 x U5 tri-snRNP complex	T, P, C
MIPS-133.40	Srb10p complex	T, P, C
GO:0005732	small nucleolar ribonucleoprotein complex	T, P, C
GO:0000119	mediator complex	T, P, C
MIPS-510.40.20	Kornberg's mediator (SRB) complex	T, P, C
MIPS-410.35	Replication complex	T, P, C

Continued on next page

Complex	Name	Experiments
EBI-1252538	Anaphase-Promoting Complex variant 3	T, P, C
GO:0016585	chromatin remodeling complex	T, P, C
GO:0000220	vacuolar proton-transporting V-type ATPase, V0 domain	T, P
MIPS-510.190.60	SBF complex	T, P, C
GO:0005846	nuclear cap binding complex	P
MIPS-90.30	ER assembly complex	T, P, C
MIPS-510.190.150	Sin3 complex	T, P, C
MIPS-230.20.20	SAGA complex	T, P, C
GO:0016580	Sin3 complex	T, P, C
MIPS-310	Nuclear pore complex (NPC)	T, P, C
GO:0008540	proteasome regulatory particle, base subcomplex	T, P, C
EBI-1251060	SAGA complex	T, P, C
MIPS-310.40	NUP84 complex	P, C
MIPS-475.10	Sister chromatid separation complex	C

Kinetochore complexes

Figure S3 lists the gene names and symbols of the genes annotated at the condensed nuclear chromosome kinetochore complex (GO:0000778) and the kinetochore complex (GO:0000776). This figure contributes in the annotation of Figure 3 of the main manuscript.

Common Name	Systematic Name
**Inner complex**	
HIR1	YBL008W
CAC1	YPR018W
MIF2	YKL089W
SGT1	YOR057W
CBF1	YJR060W
**CBF3**	
CBF3	YMR168C
CTF13	YMR094W
CEP3	YMR168C
SKP1	YDR328C
CBF2(NCD10)	YGR140W

**Central complex**	
**IPL1**	
BIR1	YJR089W
IPL1	YPL209C
SLI15	YBR156C
**CTF19**	
NKP1	YDR383C
NKP2	YLR315W
CTF3	YLR381W
MCM22	YJR135C
MCM16	YPR046W
IML3	YBR107C
CHL4	YDR254W
COMA/C1	
CTF19	YPL018W
MCM21	YDR318W
OKP1	YGR179C
AME1	YBR211C
**NDC80**	
SPC25	YER018C
SPC24	YMR117C
NUF2	YOL069W
NDC80	YIL144W
**MIND/M1**	
DSN1	YIR010W
NSL1	YPL233W
NNF1	YJR112W
MTW1	YAL034W-A

Common Name	Systematic Name
**Outer complex**	
DAM1	YGR113W
SPC19	YDR201W
DAD2	YKR083C
DAD1	YDR016C
DUO1	YGL061C
ASK1	YKL052C
SPC34	YKR037C
DAD4	YDR320C-A
DAD3	YBR233W-A

**MAPs**	
CIN8	YEL061C
KIP1	YBL063W
KIP3	YGL216W
MPS1	YDL028C
STU2	YLR045C
SLK19	YOR195W
PLC1	YPL268W
BIK1	YCL029C
BIM1	YER016W
GLE2	YER107C
**Spindle assembly checkpoints**	
MAD2	YJL030W
BUB1	YGR188C
BUB3	YOR026W
MAD1	YGL086W
MAD3	YJL013C

Figure S3: The kinetochore complexes and sub-units (in gray cells): common and systematic gene names

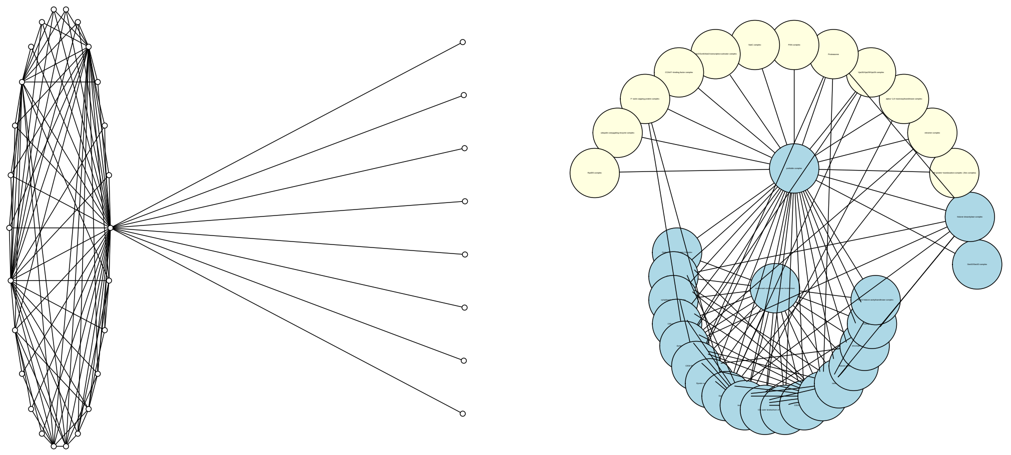

(A) (B)

Figure S4: Network of synthetic lethal interactions between prefoldin and its interacting partner. In this graph, each node represents one multi-protein complex. Panel (A) (circo layout) shows that the majority of the multi-protein complexes that interact with prefoldin are also tightly interconnected. Only 12 complexes interact solely with prefoldin. Panel (B) (twopi layout) emphasizes the role of the kinetochore complexes and its interacting partners (blue).

Prefoldin

Our results show that the prefoldin complex is among the highest interacting complexes. In fact, 50 complexes interact more than expected by chance with prefoldin (adjusted P-value ≤ 0.001). Prefoldin is a heterohexameric chaperone protein where each protein is present in equal ratios. This multi-protein complex is believed to deliver unfolded polypeptides to the chaperonin TriC complex (chaperonin-containing T-complex) and is especially involved in the biogenesis of actin and of alpha- and beta-tubulin. In fact Lacefield *et al.* [15] demonstrated that mutations in genes encoding components of the prefoldin complex result in an imbalance between alpha- and beta-tubulin which can compromise the mitotic spindles biogenesis. In our analysis, we found that prefoldin presents synthetic lethal interactions with several kinetochore complexes and spindle mitotic checkpoints. In their review Tan *et al.* [14] clearly show the complex interactions between the kinetochores and the mitotic spindles checkpoints which insure the normal chromosome segregation when the cell presents some spindle defects. Similarly, we show that those complexes not only interact with prefoldin but are also tightly inter-connected (Table S10 and Figure S4). Table S10 shows that all the kinetochore sub-complexes are tightly connected with the mitotic spindles checkpoints which emphasized their critical role as noted in the main manuscript. In Figure S4 Panel A, we show that only a few multi-protein complexes genes are not part of the highly connected kinetochores network. Panel B highlights the importance of each kinetochores sub-complexes (lower blue nodes). Finally, those results allow us to explain most of the observed synthetic lethal interaction between prefoldin, the kinetochore complexes, and the spindle checkpoints.

	AT	AI	OT	OI	Name	Size
GO:0016272	21	24	21	15	prefoldin complex	6
GO:0000778	21	14	22	5	condensed nuclear chromosome kinetochore	33
GO:0000776	16	10	16	5	kinetochore	20
GO:0031390	12	9	12	3	Ctf18 RFC-like complex	7
MIPS-270.20	7	7	7	0	Outer Kinetochor Protein Complex	21
GO:0005871	15	4	15	2	kinesin complex	6
MIPS-270.20.20	3	3	3	0	Ctf3 protein complex	3
GO:0000817	2	2	2	0	COMA complex	4
GO:0000942	2	2	2	0	outer kinetochore of condensed nuclear chromosome	2
MIPS-270.20.10	2	2	2	0	Ctf19 protein complex	3
GO:0000812	11	1	11	0	SWR1 complex	13
GO:0045298	4	1	4	0	tubulin complex	3
GO:0000123	5	1	5	0	histone acetyltransferase complex	9
GO:0005678	3	1	3	0	chromatin assembly complex	3
GO:0035267	5	1	5	0	NuA4 histone acetyltransferase complex	13
GO:0005868	16	0	16	1	cytoplasmic dynein complex	4
GO:0005869	12	0	12	1	dynactin complex	4
MIPS-140.30.30.20	12	0	12	1	Dynein-complex motorproteins	3
MIPS-140.30.30.30	12	0	12	1	Dynactin complex	3
GO:0000118	24	0	24	1	histone deacetylase complex	18

Table S10: Synthetic genetic interations between the multi-protein complexes associated with prefoldin (i.e., synthetic lethal) and the spindle assembly or the orientation cellular checkpoints. AT: munber of tested pairs with the spindle assembly checkpoint; AI: munber of pairs that interact with the spindle assembly checkpoint; OT: munber of tested pairs with the spindle orientation checkpoint; OI: munber of pairs that interact with the spindle orientation checkpoint; Descr: full name of the complex, Size: number of genes in the complex

Complex-based approach *versus* pathway-based models

We compared the results we obtained using Tong *et al.* data [10] with those published by Kelley and Ideker [3], and Ulitsky and Shamir [4]. Those authors highlighted that some of their pathways of interest are in fact multi-protein complexes. However, as detailed in the *Data sources* section our approaches slightly differ on the methods and concepts. Nevertheless we were able to evaluate whether our findings overlap.

Only Kelley and Ideker [3] investigated within-pathways relationship. We found that among the 91 within-pathways they identified, 71 are composed of genes that overlap with one or more multi-protein complexes of our interactome. This corresponds to 20 unique multi-protein complexes; none of which were found in our statistical analysis as presenting more synthetic lethal interaction that expected by chance (Table S11). These results are mainly due to the fact that we have different coverage of genetic interaction data (we only use Tong *et al.* data [10] whereas they use in addition to those data the synthetic lethal interactions catalogued at the MIPS database). Moreover the overlap between their pathways and our protein complexes is small (see the *Data sources* section for details).

Name	Complex	Size	Overlap	Pathway Size	Pathway ID
Anaphase-Promoting Complex variant 1	EBI-1252323	13	5	5	221
Anaphase-Promoting Complex variant 2	EBI-1252490	14	5	5	221
Anaphase-Promoting Complex variant 3	EBI-1252538	14	5	5	221
Anaphase-Promoting Complex variant 4	EBI-1252572	14	5	5	221
exocyst	GO:0000145	8	6	6	240
proteasome complex	GO:0000502	11	3	4	71
anaphase-promoting complex	GO:0005680	16	5	5	221
spliceosome	GO:0005681	30	3	4	228
DNA-directed RNA polymerase I complex	GO:0005736	14	4	4	485
ribosome	GO:0005840	97	4	5	429
Golgi transport complex	GO:0017119	8	2	9	401
CCR4-NOT core complex	GO:0030015	7	5	5	281
box C/D snoRNP complex	GO:0031428	50	3	4	455
small subunit processome	GO:0032040	41	3	4	455
Exocyst complex	MIPS-160	7	6	6	240
Clathrin-associated protein (AP) complex	MIPS-260.20	21	2	9	401
Nuclear pore complex (NPC)	MIPS-310	24	6	7	394
-	MIPS-310	24	5	5	166
-	MIPS-310	24	4	6	297
Nop56p/Nop1p complex	MIPS-440.12.30	3	3	4	455
CCR4 complex	MIPS-510.190.110	13	5	5	281
Anaphase promoting complex (APC)	MIPS-60	11	5	5	221

Table S11: Complex-based approach *versus* Kelley and Ideker's high density models (2005). Overlap between the content of Kelley and Ideker's pathway and the multi-protein complexes of our interactome. Name: name of the protein complex in our interactome; Complex: complex ID in our interactome; Size: size of the multi-protein complex in our interactome; Overlap: number of genes in commun between the pathway module and the overlapping multi-protein complex; Pathway Size: size of the pathway estimated by Kelley and Ideker; ID: Kelley and Ideker pathway IDs as available in their supplemental material.

In their work, Kelley and Ideker [3] also characterize 360 between-pathway interactions (we found 86). Among those 360 between-pathway interactions, we found that 236 have no pathway overlapping with our multi-protein complexes (case 1) and 110 have only one of the 2 pathways overlapping with a multi-protein complex (case 2). This leaves us with 14 between-pathway interactions where each pathway overlap with a multi-protein complex (case3 - see the *Data sources* section for details the analysis steps). We next considered those pairs of complexes and search if they share synthetic lethal interactions as reported by Tong *et al.* [10].

Among those 14 pairs, 3 correspond to 1 multi-protein complex: the 2 pathways of the between-pathway models 4460, 4947, and 4828 overlap with the nuclear pore protein complex (MIPS-310). This suggests that their pathways are not unique and that some correspond to within multi-protein complex interactions. Among the 11 remaining pairs of multi-protein complexes, we found 9 pairs that share synthetic genetic interactions as observed by Tong *et al.* [10]. Table S12 shows that 6 pairs present more interactions that expected by chance (Bonferroni adjusted P-value ≤ 0.001).Table S13 maps the interacting pairs of multi-protein complexes to the pathway IDs given by Kelley and Ideker [3].

Complex 1 - Complex 2	P-values (adjusted)	Odds	Expected	Interact	Tested	Essential Genes Complex 1	Essential Genes Complex 2
MIPS-260.20–GO:0032045	1.94e−06	67.26	0.13	6	18	3	4
GO:0000508–GO:0000118	2.23e−06	19.54	0.52	9	71	0	0
GO:0032045–GO:0030904	1.03e−05	537.84	0.04	4	5	4	0
GO:0032045–GO:0017119	1.03e−05	537.84	0.04	4	5	4	3
GO:0032045–GO:0031417	2.80e−04	Inf	0.02	3	3	4	0
GO:0043529–GO:0032045	2.80e−04	Inf	0.02	3	3	0	4
GO:0032045–GO:0008023	1.19e−01	33.61	0.11	3	15	4	4
MIPS-510.190.60–GO:0000508	5.57e−01	67.20	0.04	2	6	0	0
GO:0031390–GO:0008023	1.00e+00	11.20	0.29	3	39	4	4

Table S12: Multi-protein complexes between synthetic genetic interactions and Kelley and Ideker pathway model. P-value: Bonferroni adjusted P-value of the Hypergeometric test; OddsRatio: odds ratios; expected: expected number of synthetic genetic interaction between multi-protein complexes; tested: number of interaction tested; interact (observed): number of synthetic genetic interactions observed; Essential: number of essential genes in complex 1 and 2, respectively.Note that when all the tested interactions are found synthetic genetic interactions (tested = interact) the odds ratio are infinite (Inf).

Complex 1 - Complex 2	Names Complex 1	Names Complex 2	Pathway ID
MIPS-260.20–GO:0032045	Clathrin-associated protein (AP)	guanyl-nucleotide exchange factor	556
GO:0000508–GO:0000118	Rpd3L	histone deacetylase	2195
GO:0032045–GO:0030904	guanyl-nucleotide exchange factor	retromer	420
GO:0032045–GO:0017119	guanyl-nucleotide exchange factor	Golgi transport	556
GO:0032045–GO:0031417	guanyl-nucleotide exchange factor	NatC	504
GO:0043529–GO:0032045	GET	guanyl-nucleotide exchange factor	407
GO:0032045–GO:0008023	guanyl-nucleotide exchange factor	transcription elongation factor	634
MIPS-510.190.60–GO:0000508	SBF	Rpd3L	2224
GO:0031390–GO:0008023	Ctf18 RFC-like	transcription elongation factor	603

Table S13: Between complex-based approach versus between pathway-based approach. Identifiers and names of the multi-protein complexes map to between-pathway model IDs reported by Kelley and Ideker in their supplemental material.

Ulitsky and Shamir [4] only reported between-pathway interactions. They defined 140 between-pathway interactions of which 65 have none of their pathways overlapping with any multi-protein complex and 59 have only one of their 2 pathways overlapping with a multi-protein complex. Among the 16 pairs of pathways, 7 map more than one pair of multi-protein complexes, which reflects the content of complex variants (e.g., anaphase-promoting complex variant1 to 3 in IntAct) and overlaps in sources (GO, MIPS, and IntAct). Indeed, for some multi-protein complexes, those databases have slightly different definition of their contents. Finally, 6 pairs of multi-protein complexes share synthetic genetic interactions as observed by Tong *et al.* [10]. Table S14 shows that 5 pairs of them share more synthetic genetic interactions that expected by chance (Bonferroni adjusted P-value$\leq$ 0.001). Table S15 maps the interacting pairs of multi-protein complexes to the pathway IDs given by Ulitsky and Shamir [4].

						Essential Genes	
Complex 1 - Complex 2	P-values (adjusted)	Odds	Expected	Interact	Tested	Complex 1	Complex 2
GO:0008023–GO:0000508	1.34e−21	73.97	0.35	17	48	4	0
GO:0000778–GO:0031390	5.89e−14	59.88	0.29	12	39	19	4
GO:0030904–GO:0016272	5.93e−09	63.34	0.18	8	25	0	0
GO:0016272–GO:0000136	4.16e−08	46.81	0.23	8	31	0	0
GO:0031417–GO:0016272	4.31e−05	67.25	0.11	5	15	0	0
GO:0031390–GO:0008023	1.00e+00	11.20	0.29	3	39	4	4

Table S14: Multi-protein complexes between synthetic genetic interactions and Ulitsky and Shamir pathway model. P-value: Bonferroni adjusted P-value of the Hypergeometric test; OddsRatio: odds ratios; expected: expected number of synthetic genetic interaction between multi-protein complexes; tested: number of interaction tested; interact (observed): number of synthetic genetic interactions observed; Essential: number of essential genes in complex 1 and 2, respectively.

	Names		
Complex 1 - Complex 2	Complex 1	Complex 2	Pathway ID
GO:0008023–GO:0000508	transcription elongation factor	Rpd3L	126
GO:0000778–GO:0031390	condensed nuclear chromosome kinetochore	Ctf18 RFC-like	35
GO:0030904–GO:0016272	retromer	prefoldin	16
GO:0016272–GO:0000136	prefoldin	alpha-1,6-mannosyltransferase	17
GO:0031417–GO:0016272	NatC	prefoldin	20
GO:0031390–GO:0008023	Ctf18 RFC-like	transcription elongation factor	4

Table S15: Between complex-based approach versus between pathway-based approach. Identifiers and names of the multi-protein complexes map to between-pathway model IDs reported by Ulitsky and Shamir in their supplmental material.

Except for the interaction between the Ctf18 RFC-like complex (GO:0031390) and the transcription factor elongation complex (GO:0008023) (which does not interact more that expected by chance according to our analysis), the overlaps are different between Kelley and Ideker [3] and Ulitsky and Shamir [4] (Table S12-Table S15). We also note that among the pairs in common with Kelley and Ideker [3], the guanyl-nucleotide exchange factor (GO:0032045) appears in several interactions but not all sharing more interactions that expected by chance according to our analysis. As in our main results, Ulitsky and Shamir [4] seem to have identify the prefoldin complex (GO:0016272).

Kelley and Ideker [3] also show in Figure 2a of their paper that the prefoldin complex highly interacts with the Dynactin complex and some proteins of the kinetochore, which we also found. However we were not able to map those interactions using their supplemental material as our pathway contents differ. For instance, what they call the kinetochore in that figure is for us the Ctf19 protein complex (sub-complex of the

kinetochore). In addition, many of their pathways overlap. When using our second method of comparison (from our pairs of interacting multi-protein complexes to their pathway pairs), we found that the prefoldin complex (GO:0016272) maps 9 of their pathways, which only differ by a couple of genes (data not shown). This observation is also true for other multi-protein complexes such as the Rpd3L complex or GO:0000508 (which maps 12 pathways) or the transcription elongation factor or GO:0008023 (which maps 6 pathway). Similarly, in Ulitsky and Shamir [4] work, the prefoldin complex overlaps with 10 of their pathways.

Using our second method of comparison, we also found pairs of multi-protein complexes mapping pathways that Kelley and Ideker [3] did not find to interact. For example, we found that the Holliday junction resolvase (GO:0048476) and the RecQ helicase topo III complex (GO:0031422) highly interact (P-value$= 1.12e - 10$). In addition, Fujikane *et al.* [28] demonstrate that the Holliday junction resolvase has a RecQ-like function hence may substitute to RecQ. The Holliday junction resolvase complex maps pathwayID 393 and the RecQ helicase topo III complex overlaps with pathwayID 79. However, according to Kelley and Ideker, those pathway do not interact. This result can be explained by the fact those complexes are small (2 proteins for GO:0048476 and 3 for GO:0031422) and few interactions were tested but all were positive (see TableS6-long-Tong.csv file). This points out an other limit of the density-based approach proposed by Kelley and Ideker [3].
